# Supplementary material for: AI-enhanced integration of genetic and medical imaging data for risk assessment of Type 2 diabetes
Source: Nat Commun. 2024 May 18;15:4230. doi: 10.1038/s41467-024-48618-1 (PMC11102564; doi:10.1038/s41467-024-48618-1)
Supplement: Supplementary file 1 — Supplementary Information [file 41467_2024_48618_MOESM1_ESM.pdf]

Supplemental information file of the paper entitled "AI-Enhanced Integration of Genetic and Medical Imaging Data for Risk Assessment of Type 2 Diabetes"

Yi-Jia Huang[1], Chun-houh Chen[2], and Hsin-Chou Yang[1,2,3,4,*]

[1]Institute of Public Health, National Yang-Ming Chiao-Tung University, Taipei, Taiwan
[2]Institute of Statistical Science, Academia Sinica, Taipei, Taiwan
[3]Biomedical Translation Research Center, Academia Sinica, Taipei, Taiwan
[4]Department of Statistics, National Cheng Kung University, Tainan, Taiwan

*Corresponding author: Hsin-Chou Yang, Institute of Statistical Science, Academia Sinica. No. 128, Sec. 2, Academia Road, Nankang 115, Taipei, Taiwan

(Fax) 886-2-27886833
(Tel) 886-2-27875686
(E-mail) hsinchou@stat.sinica.edu.tw

Supplemental Information

Figure S1	Sample sizes in the two analyses in this study……………………………	3
Figure S2	Performance of the SNP predictors selected from published GWAS or our GWAS with different p-value thresholds………………	4
Figure S3	The T2D family history's performance combined with other demographic and genetic predictors………………………………………	5
Figure S4	Manhattan plot of genome-wide association study……………………	6
Figure S5	Prediction AUCs of all the models……………………………………………	7
Figure S6	Classification AUCs of all the models……………………………………….	8
Figure S7	Identification of high-risk groups……………………………………………..	9
Figure S8	Risk factors for T2D…………………………………………………………………….	10
Figure S9	Effect of doing exercise on HbA1c……………………………………………	11
Figure S10	ROC plots and the corresponding AUC for the models considering the combinations of image report variables, genetic factors, and demographic factors…………………………………………….	12
Figure S11	Cumulative and incremental AUCs of the top feature variables in the best model…………………………………………………………………………..	13
Table S1	Data used in the two analyses in this study……………………………..	14
Table S2	Performance evaluation for the models that add environmental factors or SNP x SNP interactions as predictors………………………..	15
Table S3	Cox regression analysis with different considerations of time	

	scales and sex variable treatments……………………………………………	16
Table S4	Comparison between prediction models using default parameters and tuned parameters……………………………………………..	17
Supp Text 1	Input data for an online T2D-risk assessment…………………………	18
Supp Text 2	Sources of genetic variable data………………………………………………	19
References	……………………………………………………………………………………………	20

Supplementary Figures

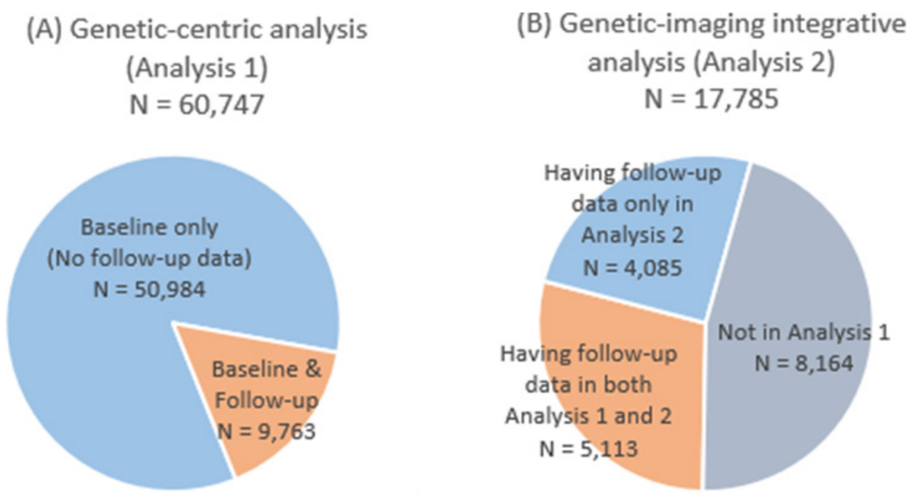

Figure S1. Sample sizes in the two analyses in this study. (A) Analysis 1: Genetic-centric analysis. Total number of participants: N = 68,911 (= 50,984 + 9,763 + 8,164). The genetic-centric analysis (Analysis 1) included 60,747 participants, including 50,984 participants with only baseline data and 9,763 participants with both baseline and follow-up data. **(B) Analysis 2: Genetic-imaging integrative analysis.** The genetic-imaging integrative analysis (Analysis 2) included a total of 17,785 participants who had baseline data, follow-up data, and image data, of which 9,198 (= 4,085 + 5,113) participants were included in Analysis 1, and additional 8,164 participants were only included in Analysis 2. The unique participants in the two analyses in this study were N = 68,911 (= 50,984 + 9,763 + 8,164).

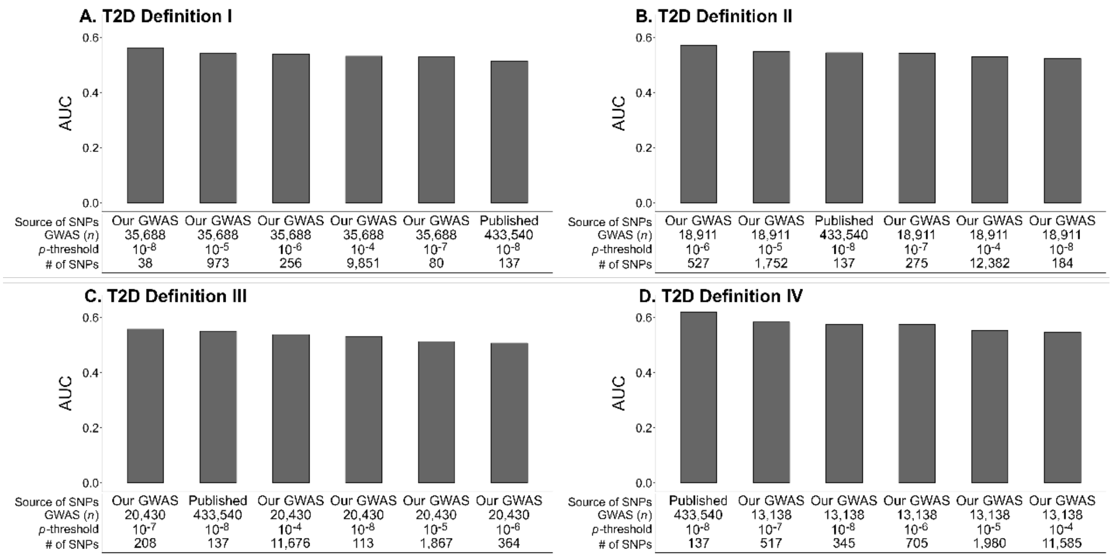

Figure S2. Performance of the SNP predictors selected from published GWAS or our GWAS with different p-value thresholds. Model predictors were SNPs selected from published studies or our GWAS under different p-value thresholds, where our GWAS association test is a two-sided Wald test for the slope coefficient in a logistic regression. AUC is displayed via a bar chart. AUCs of different SNPs are listed from high to low. **(A) T2D Definition I** (N = 35,688); **(B) T2D Definition II** (N = 18,911); **(C) T2D Definition III** (N = 20,430); **(D) T2D Definition IV** (N = 13,138). In general, the difference in AUCs is limited. Source data are provided as a Source Data file.

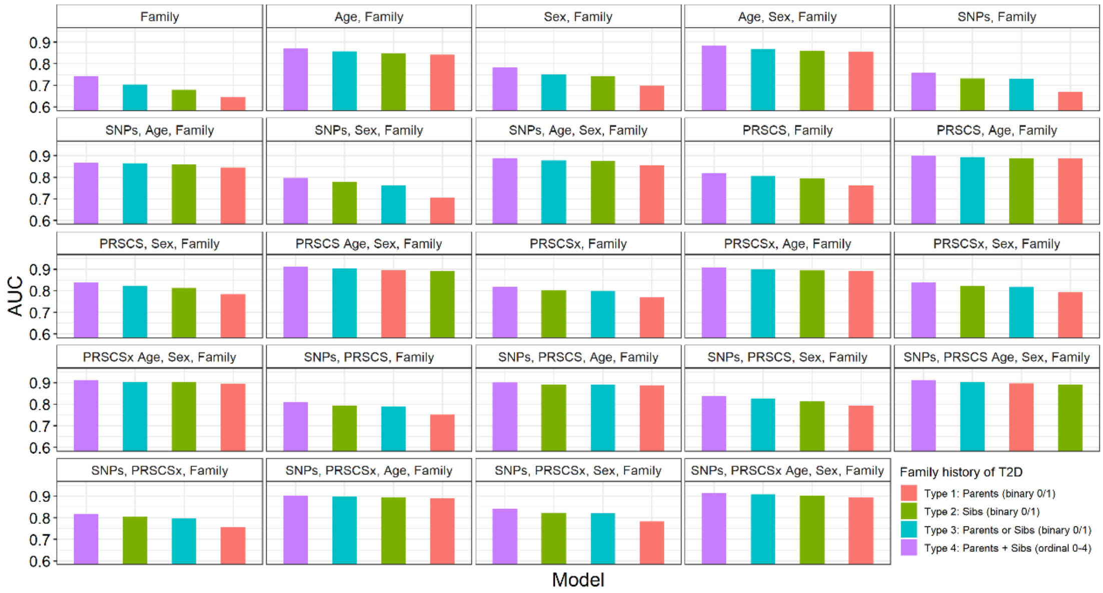

Figure S3. The T2D family history's performance combined with other demographic and genetic predictors. The model considering both parents' and sibs' T2D history (purple bars) outperforms other types of T2D family history. Source data are provided as a Source Data file.

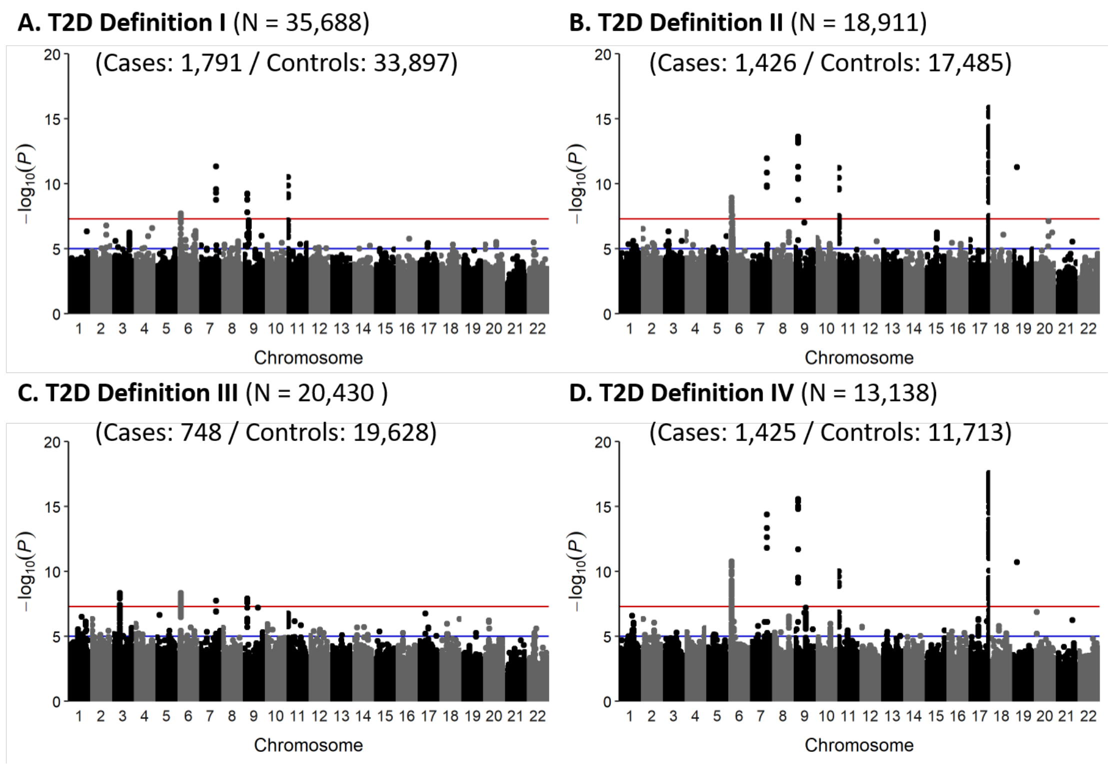

Figure S4. Manhattan plot of genome-wide association study. T2D-associated SNPs, HbA1c, and fasting glucose were identified. **(A) T2D Definition I**; **(B) T2D Definition II**; **(C) T2D Definition III**; **(D) T2D Definition IV**. Source data are provided as a Source Data file.

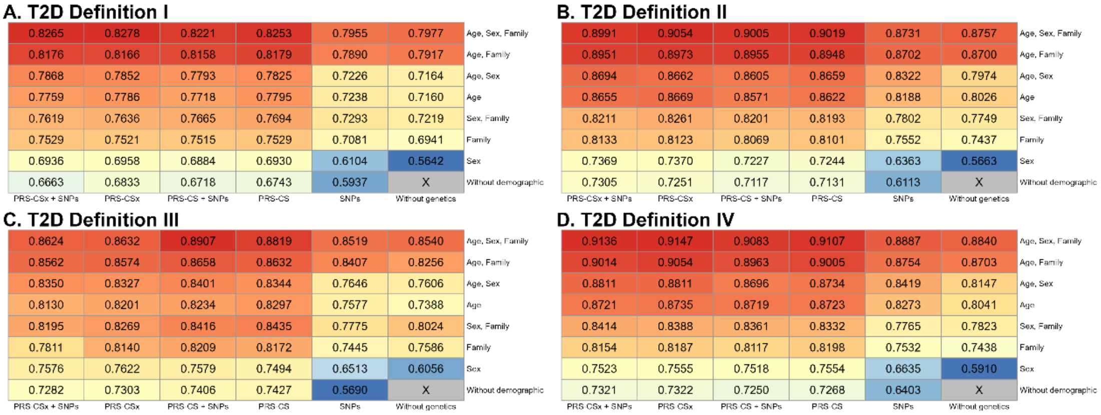

Figure S5. Prediction AUCs of all the models. A heatmap shows the models' prediction AUCs in four T2D definitions. The genetic variables are shown on the X-axis, and the demographic variables are shown on the Y-axis. **(A) T2D Definition I**; **(B) T2D Definition II**; **(C) T2D Definition III**; **(D) T2D Definition IV**. Source data are provided as a Source Data file.

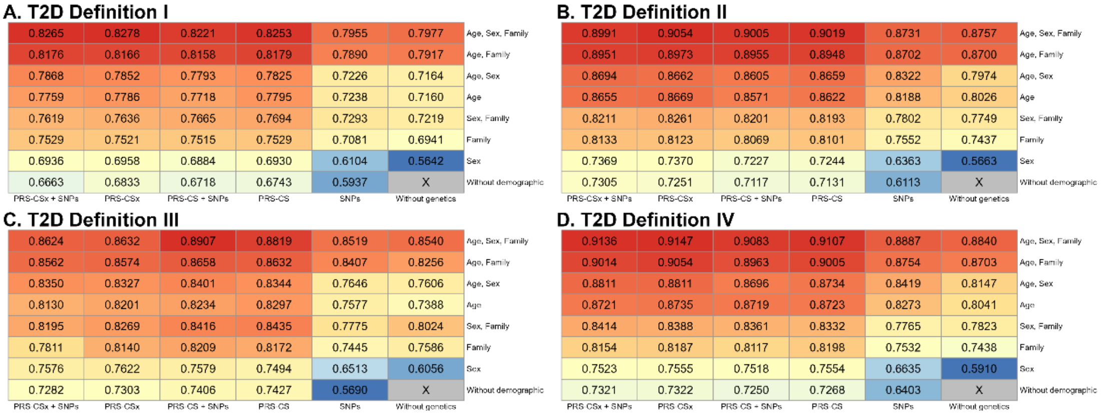

Figure S6. Classification AUCs of all the models. A heatmap shows the models' classification AUCs in four T2D definitions. The genetic variables are shown on the X-axis, and the demographic variables are shown on the Y-axis. **(A) T2D Definition I**; **(B) T2D Definition II**; **(C) T2D Definition III**; **(D) T2D Definition IV**. Source data are provided as a Source Data file.

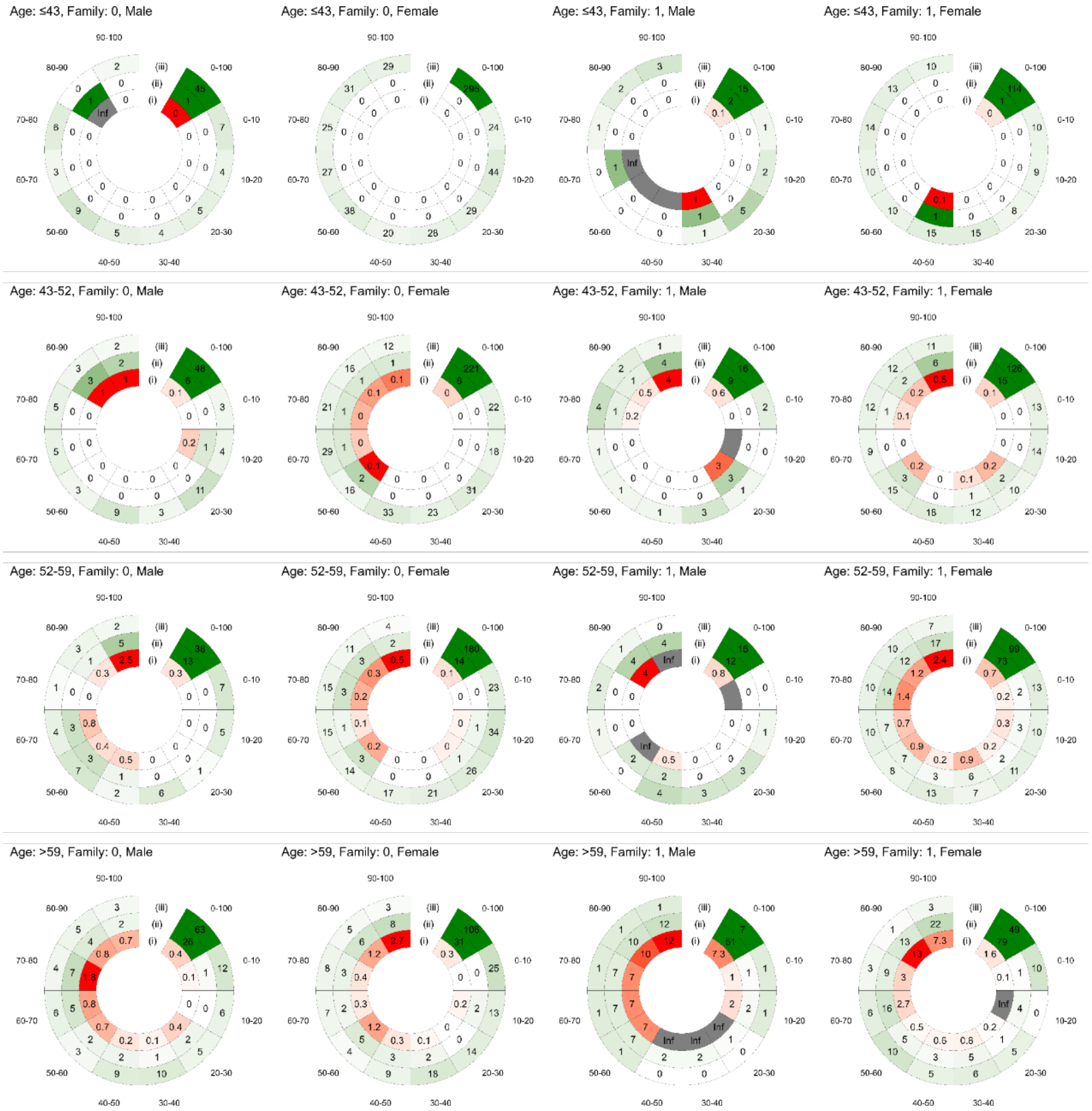

Figure S7. Identification of high-risk groups. A stratified analysis across various combinations of age subgroups, sex subgroups, and family history subgroups was performed to identify high-risk subgroups. In each chart, the figures from the inner to the outer represent (i) the case-to-control ratio, (ii) the number of cases, and (iii) the number of controls in the PRS decile subgroups. Source data are provided as a Source Data file.

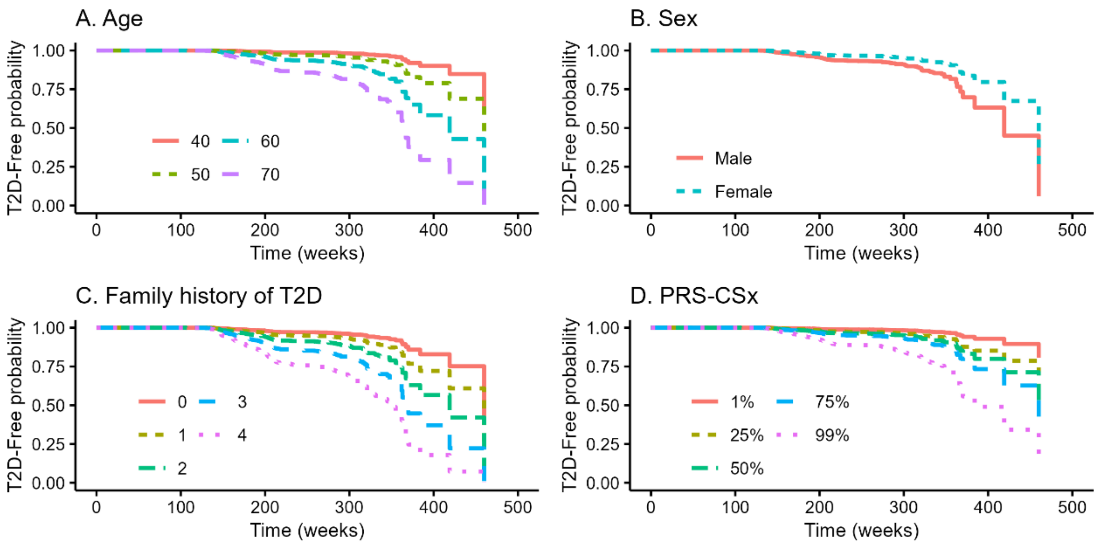

Figure S8. Risk factors for T2D. Kaplan-Meier curves reveal significant T2D risk factors (high-risk level). **(A) Age (older persons)**; **(B) Sex (males)**; **(C) T2D family history (the number of parents and siblings who had T2D)**; and **(D) PRS (high decile PRS group)**. Source data are provided as a Source Data file.

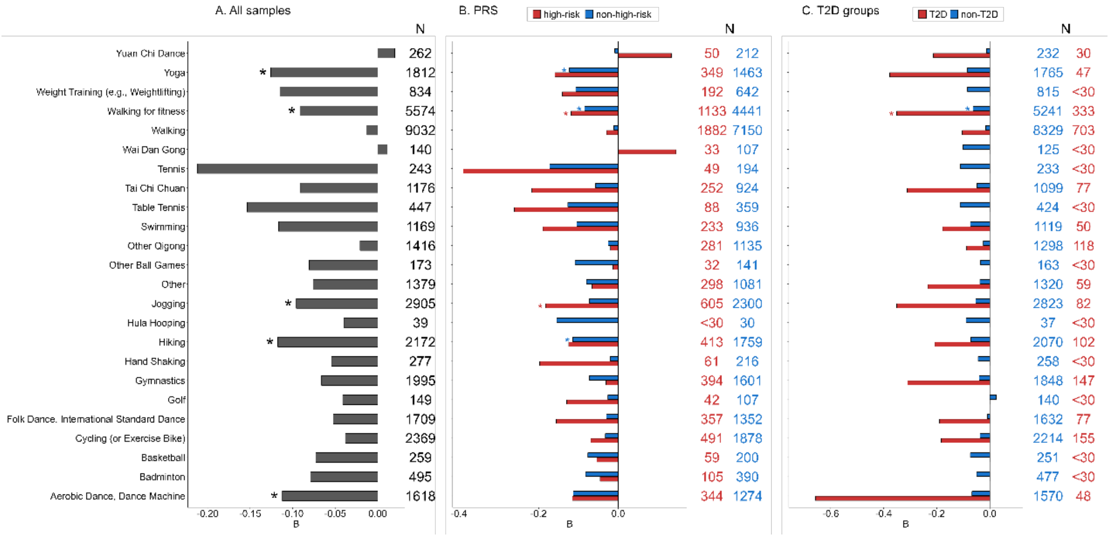

Figure S9. Effect of doing exercise on HbA1c. Using a linear regression, slope coefficients (*B*) for various types of exercise are displayed via a bar chart. In total, N = 59,811 samples; the figures on the right-hand side indicate the number of individuals doing each type of exercise. Only the result of an analysis containing a sample size >30 is shown. **(A) All samples**; **(B) PRS.** Beta coefficients for the high-risk group (red bar) and non-high-risk group (blue) are displayed; **(C) T2D groups**. Beta coefficients for the T2D group (red bar) and non-T2D group (blue) are displayed. Walking for fitness was found to be significantly negatively associated with HbA1c in all samples, including high and low-risk subgroups, and both T2D and non-T2D groups. Source data are provided as a Source Data file.

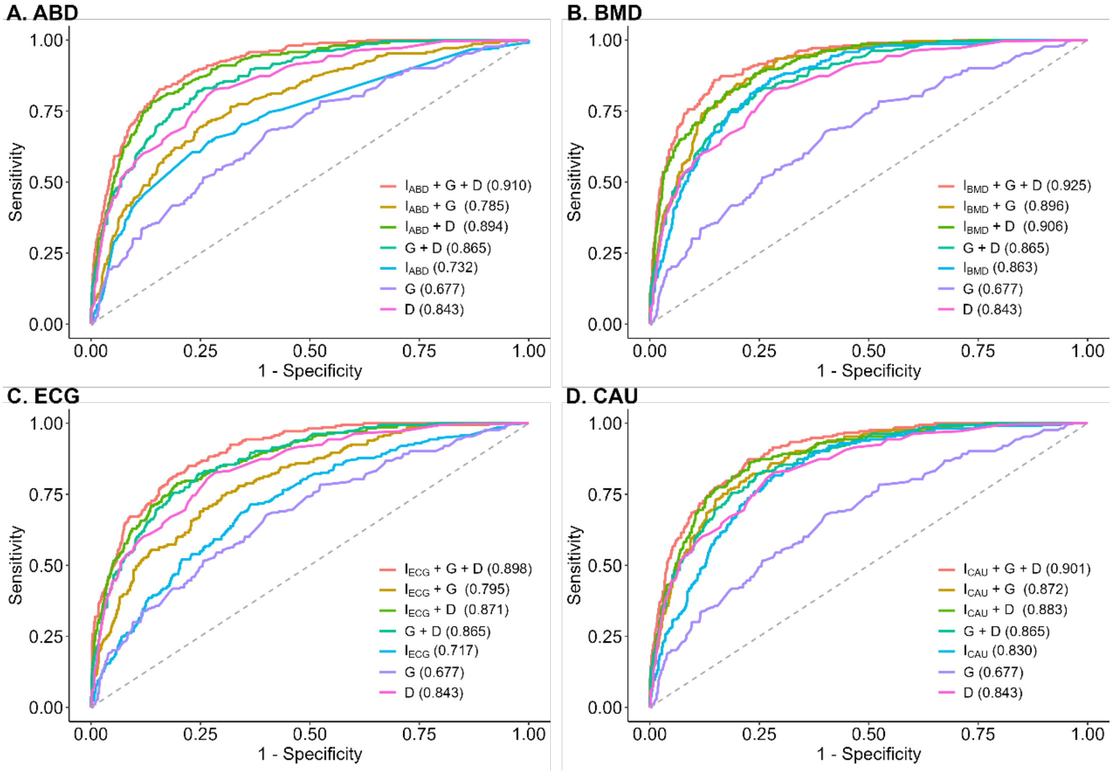

Figure S10. ROC plots and the corresponding AUC for the models considering the **combination of image report variables, genetic factors, and demographic factors.** ROC plots and the corresponding AUC for the models considering medical image features (I), genetic PRS (G), and demographic variables, including age, sex, T2D family history (D), and their combinations. **(A) ABD features**; **(B) BMD features**; **(C) ECG features**; **(D) CAU features**. BMD had the best performance. Source data are provided as a Source Data file.

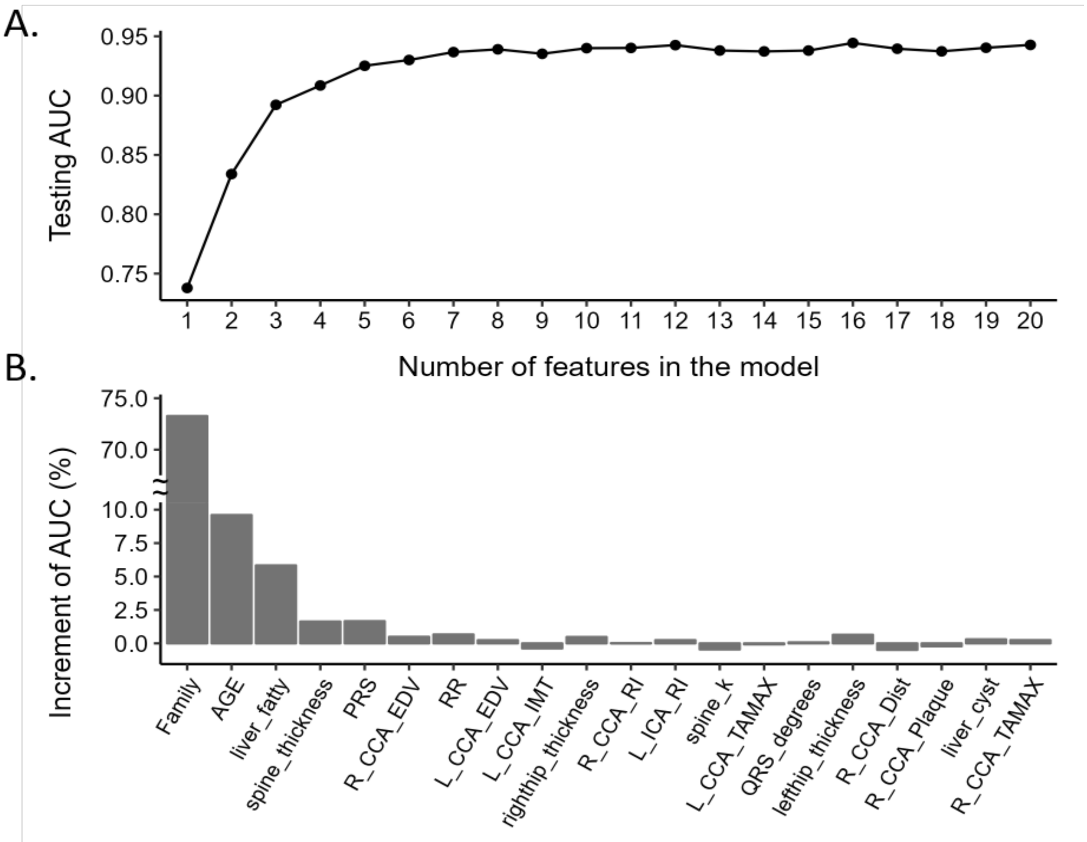

Figure S11. Cumulative and incremental AUCs of the top feature variables in the best model. (A) Cumulative AUC. The best prediction model, which contains age, family history of T2D, PRS, and 125 medical imaging feature variables, achieves an AUC of 0.949. In this figure, we list the top 20 feature variables in the best model. The reduced model, comprising only the top eight essential variables – family history (from the questionnaire), age (from the questionnaire), fatty liver (from ABD images), spine thickness (from BMD images), polygenic risk score (PRS) (from genetic data), end-diastolic velocity in the right common carotid artery (R_CCA_EDV) (from CAU images), RR interval (from ECG images), and end-diastolic velocity in the left common carotid artery (L_CCA_EDV) (from CAU images) – maintains a commendable AUC of 0.939. **(B) Incremental AUC (%) for each of the top feature variables.** Feature variables are sorted based on the importance of the feature in the XGBoost model (see Fig. 6C). Family history is the most crucial feature, with an AUC of 0.738. Conditional on family history in the model, the inclusion of age results in an increase of the total AUC to 0.834, with an increment of AUC of 0.096. Subsequent sequential inclusion of fatty liver (from ABD images), spine thickness (from BMD images), polygenic risk score (PRS) (from genetic data), end-diastolic velocity in the right common carotid artery (R_CCA_EDV) (from CAU images), RR interval (from ECG images), and end-diastolic velocity in the left common carotid artery (L_CCA_EDV) (from CAU images) yields total AUC values of 0.892, 0.909, 0.925, 0.930, 0.937, and 0.939 with increment values of 0.058, 0.016, 0.017, 0.005, 0.006, and 0.002, respectively. Source data are provided as a Source Data file.

Supplementary Tables

Table S1. Data used in the two analyses in this study. TWB data comprise data in the baseline and the follow-up. In the genetic-centric analysis (Analysis 1), classification models were built and tested based on the variables and phenotype data in the baseline. Prediction models were built based on the variables and phenotype data in the baseline. The model was tested based on the prediction variables in the baseline and T2D phenotype in the follow-up. In the genetic-imaging integrative analysis (Analysis 2), classification models were built and tested based on the predictors and phenotype data in the follow-up and further replicated based on the second independent testing dataset.

	Baseline	Follow-up (①Early / ②Late)
**TWB Data**	Genotyping data (TWB1.0, TWB2.0)<br>Questionnaire (Self-reported disease, Demo., Family history, Enviro.)<br>Blood and urine tests (HbA1c, GLU-AC)	① / ② Medical images (ABD, BMD, EKG, VAS, TU)<br>① / ② Questionnaire (Self-reported disease, Demo., Family history, Enviro.)<br>① / ② Blood and urine tests (HbA1c, GLU-AC)

Analysis 1 – Genetic-centric analysis

			Baseline	Follow-up	Sample size
Classification	Mode building	Phenotype	Self-reported T2D status<br>HbA1c, GLU-AC		50,984
		Predictors	TWB2.0 imputed<br>Demo., Family history, Enviro.		
	Mode testing	Phenotype	Self-reported T2D status<br>HbA1c, GLU-AC		8,827
		Predictors	TWB2.0 imputed<br>Demo., Family history, Enviro.		
Prediction	Mode building	Phenotype	Self-reported T2D status<br>HbA1c, GLU-AC		50,984
		Predictors	TWB2.0 imputed<br>Demo., Family history, Enviro.		
	Mode testing	Phenotype		① Self-reported T2D status<br>① HbA1c, GLU-AC	8,827
		Predictors	TWB2.0 imputed<br>Demo., Family history, Enviro.		
	Independent testing	Phenotype		② Self-reported T2D status<br>② HbA1c, GLU-AC	936
		Predictors	TWB2.0 imputed<br>Demo., Family history, Enviro.		

Analysis 2 – Genetic-imaging integrative analysis

			Baseline	Follow-up	Sample size
Classification	Mode building	Phenotype		① Self-reported T2D status<br>① HbA1c, GLU-AC	5,864
		Predictors	TWB2.0 imputed	① Demo., Family history, Enviro.	
	Mode testing	Phenotype		① Self-reported T2D status<br>① HbA1c, GLU-AC	1,469
		Predictors	TWB2.0 imputed	① Demo., Family history, Enviro.	
	Independent testing	Phenotype		② Self-reported T2D status<br>② HbA1c, GLU-AC	444
		Predictors	TWB2.0 imputed	② Demo., Family history, Enviro.	

Table S2. Performance evaluation for the models that add environmental factors or SNP x SNP interactions as predictors. Demographic factors (Demo.) include age, sex, and family history of T2D. Genetic information includes PRS-CSx, SNP Main effect, and SNP interactions. Environmental factors include education, drinking experience, exercise habits, and the number of exercise types. SNP-SNP interactions include 1,059 SNP pairs identified through allele-based interaction tests using Z-scores, examining differences in SNP-SNP association (odds ratio) between case and control groups ($p < 10\times10^{-10}$). Moreover, SNP's main effect includes 1,191 SNPs from 1,059 pairs of SNPs.

	Features					Performance				
	Demo.	PRS-CSx	Environmental	SNPs Main effect	SNPs Interaction	AUC	Accuracy	Sensitivity	Specificity	F1
Model 1	O	O	X	X	X	0.9147	0.8429	0.8437	0.8427	0.6722
Model 2	O	O	O	X	X	0.9146	0.8440	0.8142	0.8511	0.6659
Model 3	O	O	X	X	O	0.9111	0.7928	0.8879	0.7704	0.6206
Model 4	O	O	O	X	O	0.9125	0.7675	0.9204	0.7314	0.6017
Model 5	O	O	X	O	O	0.9044	0.7973	0.8702	0.7801	0.6211
Model 6	O	O	O	O	O	0.9112	0.7866	0.9027	0.7592	0.6176

Table S3. Cox regression analysis with different considerations of time scales and sex variable treatments. The Cox regression analysis considered two types of time scales (i.e., time-on-study and age) and three types of sex variable treatment (i.e., adjusting for sex as a covariate, conducting sex-specific analysis with an assumption of a common sex effect, and performing sex-specific analysis with different sex effects), resulting in six analyses. The initial three analyses considered time-on-study as the time scale, with age at baseline included as a covariate, and incorporating the following sex variable treatment: (1) Model 1: Sex was treated as a covariate in the analysis; (2) Model 2: Sex-specific analysis, assuming a common effect for males and females; (3) Model 3: Sex-specific analysis with different effects for males and females. The subsequent three analyses considered age as the time scale, with age-at-baseline as left truncation, along with the three sex variable treatments, similar to the time-on-study analysis, to be Models 4 – 6.

Covariates	Time scale: Time-on-study			
	**Model 1** Sex as covariate	**Model 2** Sex-specific analysis, assuming a common effect for males and females	**Model 3** Sex-specific analysis with different effects for males and females	
	HR (95% CI)			
			Male	Female
Age	1.087 (1.067, 1.106)	1.087 (1.067, 1.106)	1.057 (1.027,1.088)	1.103 (1.078,1.129)
Sex-Female	0.557 (0.419,0.741)	-	-	-
Family	1.640 (1.410, 1.907)	1.636 (1.407, 1.903)	1.534 (1.146,2.053)	1.666 (1.394,1.991)
PRS	1.643 (1.436, 1.880)	1.651 (1.442, 1.890)	1.840 (1.458, 2.322)	1.565 (1.325,1.848)

(continued).

Covariates	Time scale: Age			
	**Model 4** Sex as covariate	**Model 5** Sex-specific analysis, assuming a common effect for males and females	**Model 6** Sex-specific analysis with different effects for males and females	
	HR (95% CI)			
			Male	Female
Age	-	-	-	-
Sex-Female	0.582 (0.439, 0.773)	-	-	-
Family	1.585 (1.368, 1.836)	1.568 (1.353,1.817)	1.521 (1.140,2.029)	1.591 (1.339, 1.890)
PRS	1.659 (1.448, 1.900)	1.661 (1.449,1.904)	1.765 (1.398,2.230)	1.608 (1.359, 1.903)

Table S4. Comparison between prediction models using default parameters and tuned parameters. A grid search method was applied to optimize several parameters in the best model. The tuned parameters encompassed the learning rate (0.001, 0.01, 0.3), the minimum loss reduction required for splitting (gamma = 0, 0.1, 1, 1.5), the maximum depth of a tree (max_depth = 3, 6, 9), the minimum sum of instance weight required in a child (min_child_weight = 1, 5, 10), the subsample ratio of training instances (subsample = 0.8, 0.9, 1), and the control of the balance between positive and negative weights (scale_pos_weight = 0, 1, 2, 4). The default parameters were set as follows: learning rate = 0.3, gamma = 0, max_depth = 6, min_child_weight = 1, subsample = 1, and scale_pos_weight = 1. Out of the 1,296 training combinations, the optimal parameters were determined to be: learning rate = 0.3, gamma = 1.5, max_depth = 3, min_child_weight = 1, subsample = 0.8, and scale_pos_weight = 2.

Data	Parameter	Performance				
		AUC	Accuracy	Sensitivity	Specificity	F1-score
Testing data 1	default	0.9487	0.8707	0.8779	0.8694	0.6631
	tuned	0.9496	0.8993	0.7793	0.9196	0.6917
Testing data 2	default	0.9287	0.8536	0.7885	0.8622	0.5578
	tuned	0.9299	0.8896	0.7692	0.9056	0.6202

Supplementary Texts

Supplemental Text 1: Input data for an online T2D-risk assessment.

Users must upload their individual genotype files following the PLINK format containing bed, bim, and fam. Click “example” to download the example files. Users should upload their files to the medical imaging information following the CSV format. Click “example” to download an example file.

Supplemental Text 2: Sources of genetic variable data.

Sources of genetic variable data were considered and compared (Fig. 1D and Fig. 2B): (i) a set of SNPs selected from our GWAS; (ii) a set of highly significant T2D-associated SNPs from publications; (iii) polygenic risk score (PRS) based on the GWAS summary statistics of T2D from publications; (iv) SNP-SNP and SNP-environment interactions. They are elaborated as follows.

As to (i), GWASs for four phenotypes (Figs. 1B and 1C) were conducted using logistic regression with an adjustment for age, sex, and principal components PC1 to PC10 and an additive model of SNP. For each phenotype definition, different p-value thresholds ($p < 10^{-4}$, 10^{-5}, 10^{-6}, 10^{-7}, and 10^{-8}) were applied.

As to (ii), a set of 137 highly significant T2D-associated SNPs from the Asian Genetic Epidemiology Network (AGEN) (Spracklen, Horikoshi [1]) that is a meta-analysis based on 77,418 T2D cases and 356,122 healthy controls from 23 GWASs.

As to (iii), SNP effects were estimated by using PRS-CS [2] based on the meta-GWAS summary statistics of T2D in East Asia in the DIAGRAM Consortium [3] and the linkage disequilibrium (LD) reference from the EAS population of the 1000 Genomes Project [4]. PRS was calculated using PLINK (--score command) based on our genotype data, and 884,327 SNP effects were estimated using PRS-CS. Normalized PRS was standardized to mean = 0 and standard deviation = 1.

As to (iv), SNP effects were estimated by using PRS-CSx [5] based on the meta-GWAS summary statistics of T2D in multiple populations, including (a) East Asian of 56,268 cases and 227,155 controls in the DIAGRAM Consortium [3]; (b) European of 80,154 cases and 853,816 controls in the DIAGRAM Consortium [3]; (c) South Asian of 16,540 cases and 32,952 controls in the DIAGRAM Consortium [3], and the LD reference from each of the three populations (EAS, EUR, and SAS). 884,327, 880,098, and 900,047 SNPs for EAS, EUR, and SAS were applied to our data to calculate the population-specific PRS for each individual using the PLINK (--score command). We combined the three population-specific PRS with equal weight to calculate a final PRS. R language was used to standardize the PRS to mean = 0 and standard deviation = 1.

(v) Finally, we considered all pairwise SNP-SNP and SNP-environment interactions. SNP-SNP interactions considered all pairwise combinations of SNPs, then selected SNP-SNP interaction pairs satisfying $p < 10^{-10}$ using PLINK --fast-epistasis command.

References

1. Spracklen, C.N., et al., *Identification of type 2 diabetes loci in 433,540 East Asian individuals.* Nature, 2020. **582**(7811): p. 240-245.
2. Ge, T., et al., *Polygenic prediction via Bayesian regression and continuous shrinkage priors.* Nature Communications, 2019. **10**(1): p. 1776.
3. Mahajan, A., et al., *Multi-ancestry genetic study of type 2 diabetes highlights the power of diverse populations for discovery and translation.* Nat Genet, 2022. **54**(5): p. 560-572.
4. Auton, A., et al., *A global reference for human genetic variation.* Nature, 2015. **526**(7571): p. 68-74.
5. Ruan, Y., et al., *Improving polygenic prediction in ancestrally diverse populations.* Nature Genetics, 2022. **54**(5): p. 573-580.
