# Supplementary material for: AI-enhanced integration of genetic and medical imaging data for risk assessment of Type 2 diabetes
Source: Nat Commun. 2024 May 18;15:4230. doi: 10.1038/s41467-024-48618-1 (PMC11102564; doi:10.1038/s41467-024-48618-1)
Supplement: Supplementary file 3 — Description of Additional Supplementary Files [file 41467_2024_48618_MOESM3_ESM.pdf]

Description of Additional Supplementary Files

Supplementary Data 1 provides the description of image report features.
