# Supplementary material for: AI-enhanced integration of genetic and medical imaging data for risk assessment of Type 2 diabetes
Source: Nat Commun. 2024 May 18;15:4230. doi: 10.1038/s41467-024-48618-1 (PMC11102564; doi:10.1038/s41467-024-48618-1)
Supplement: Supplementary file 4 — Supplementary Data 1 [file 41467_2024_48618_MOESM4_ESM.pdf]

Supplementary Data 1. Description of image report features.

Variable	Description
CAU	
R_CCA_EDV	End-diastolic velocity in the right common carotid artery
R_CCA_EDV	End-diastolic velocity in the right common carotid artery
R_CCA_TAMAX	Time average maximum mean velocity of right common carotid artery
R_CCA_PI	Pulsatility Index in the right common carotid artery
R_CCA_RI	Resistive index in the right common carotid artery
R_CCA_Dist	Diameter of the right common carotid artery
R_CCA_IMT	Intima-media thickness in the right common carotid artery
R_ICA_Plaque	Plaque in the right internal carotid artery
R_ICA_PSV	Peak systolic velocity in the right internal carotid artery
R_ICA_EDV	End-diastolic velocity in the right internal carotid artery
R_ICA_TAMAX	Time average maximum mean velocity of right internal carotid artery
R_ICA_PI	Pulsatility Index in the right internal carotid artery
R_ICA_RI	Resistive index in the right internal carotid artery
R_ICA_Dist	Diameter of the right internal carotid artery
L_CCA_PSV	Peak systolic velocity in the left common carotid artery
L_CCA_EDV	End-diastolic velocity in the left common carotid artery
L_CCA_TAMAX	Time average maximum mean velocity of left common carotid artery
L_CCA_PI	Pulsatility Index in the left common carotid artery
L_CCA_RI	Resistive index in the left common carotid artery
L_CCA_Dist	Diameter of the left common carotid artery
L_CCA_IMT	Intima-media thickness in the left common carotid artery
L_ICA_Plaque	Plaque in the left common carotid artery
L_ICA_PSV	Peak systolic velocity in the left internal carotid artery

Supplementary Data 1 (continued).

Variable	Description
CAU	
L_ICA_EDV	End-diastolic velocity in the left internal carotid artery
L_ICA_TAMAX	Time average maximum mean velocity of left internal carotid artery
L_ICA_PI	Pulsatility Index in the left internal carotid artery
L_ICA_RI	Resistive index in the left internal carotid artery
L_ICA_Dist	Diameter of the left internal carotid artery
ECG	
QRS	QRS complex
QTcBaz	Corrected QT interval (Bazett's formula)
PR	PR interval
P	P wave
RR	RR interval
PP	PP interval
P_degrees	P-wave axis
QRS_degrees	QRS axis
T_degrees	T-wave axis
ECG_summary	ECG summary
BMD	
spine_k	Spine k
spine_d0	Spine d0
spine_thickness	Spine thickness
spine_ROI	Width of the region of interest in the spine
spine_ROI_length	Length of the region of interest in the spine
spine_L1_area	bone area in the lumbar spine (L1)
spine_L1_BMC	bone mineral content in the lumbar spine (L1)
spine_L1_BMD	bone mineral density in the lumbar spine (L1)
spine_L1_Tscore	lumbar spine (L1) T-Score
spine_L1_Zscore	lumbar spine (L1) Z-Score
spine_L1_peakreference	The peak reference value for the lumbar spine (L1)
spine_L1_agematched	Age-Matched value for lumbar spine (L1)
spine_L2_area	bone area in the lumbar spine (L2)
spine_L2_BMC	bone mineral content in the lumbar spine (L2)
spine_L2_BMD	bone mineral density in the lumbar spine (L2)
spine_L2_Tscore	lumbar spine (L2) T-Score

Supplementary Data 1 (continued).

Variable	Description
BMD	
spnce_L2_Zscore	lumbar spine (L2) Z-Score
spine_L2_peakreference	The peak reference value for the lumbar spine (L2)
spine_L2_agematched	Age-Matched value for lumbar spine (L2)
spine_L3_area	bone area in the lumbar spine (L3)
spine_L3_BMC	bone mineral content in the lumbar spine (L3)
spine_L3_BMD	bone mineral density in the lumbar spine (L3)
spine_L3_Tscore	lumbar spine (L3) T-Score
spine_L3_Zscore	lumbar spine (L3) Z-Score
spine_L3_peakreference	The peak reference value for the lumbar spine (L3)
spine_L3_agematched	Age-Matched value for lumbar spine (L3)
spine_L4_area	bone area in the lumbar spine (L4)
spine_L4_BMC	bone mineral content in the lumbar spine (L4)
spine_L4_BMD	bone mineral density in the lumbar spine (L4)
spine_L4_Tscore	lumbar spine (L4) T-Score
spine_L4_Zscore	lumbar spine (L4) Z-Score
spine_L4_peakreference	The peak reference value for the lumbar spine (L4)
spine_L4_agematched	Age-Matched value for lumbar spine (L4)
spine_total_area	Spine total area
spine_total_BMC	Spine total bone mineral content
spine_total_BMD	Spine total bone mineral density
spine_total_Tscore	Spine total T-Score
spine_total_Zscore	Spine total Z-Score
spine_total_peakreference	Spine total peak reference value
spine_total_agematched	Spine total age-matched value
lefthip_K	Left hip k
lefthip_d0	Left hip d0
lefthip_thickness	The thickness of the left hip
lefthip_ROI_width	Width of the region of interest in the left hip
lefthip_ROI_length	Length of the region of interest in the left hip
lefthip_neck_area	The neck area of the left hip
lefthip_neck_BMC	Neckbone mineral of the left hip
lefthip_neck_BMD	Neckbone mineral density of the left hip
lefthip_neck_Tscore	Neck T-Score of the left hip
lefthip_neck_Zscore	Neck Z-Score of the left hip
lefthip_neck_peakreference	Neck peak reference value of the left hip

Supplementary Data 1 (continued).

Variable	Description
BMD	
lefthip_neck_agematched	Neck age-matched value of the left hip
lefthip_total_area	Neck total area of the left hip
lefthip_total_BMC	Neck total bone mineral of the left hip
lefthip_total_BMD	Neck total bone mineral density of the left hip
lefthip_total_Tscore	Neck total T-Score of the left hip
lefthip_total_Zscore	Neck total Z-Score of the left hip
lefthip_total_peakreference	Neck total peak reference value of the left hip
lefthip_total_agematched	Neck total age-matched value of the left hip
righthip_K	Right hip k
righthip_d0	Right hip d0
righthip_thickness	The thickness of the right hip
righthip_ROI_thickness	Width of the region of interest in the right hip
righthip_ROI_length	Length of the region of interest in the right hip
righthip_neck_width	Neck width of the right hip
righthip_neck_area	The neck area of the right hip
righthip_neck_BMC	Neckbone mineral of the Left Hip
righthip_neck_BMD	Neckbone mineral density of the right hip
righthip_neck_Tscore	Neck T-Score of the right hip
righthip_neck_Zscore	Neck Z-Score of the right hip
righthip_neck_peakreference	Neck peak reference value of the right hip
righthip_neck_agematched	Neck age-matched value of the right hip
righthip_total_area	Neck total area of the right hip
righthip_total_BMC	Neck total bone mineral of the right hip
righthip_total_BMD	Neck total bone mineral density of the right hip
righthip_total_Tscore	Neck total T-Score of the right hip
righthip_total_Zscore	Neck total Z-Score of the right hip
righthip_total_peakreference	Neck total peak reference value of the right hip
righthip_total_agematched	Neck total age-matched value of the right hip
MEASURE_BONE_POSE	Dominant limb for bone density measurement
BONE_EXAM_RESULT	Bone Stiffness Index
YOUNG_ADULT	Percentile in young adults
T_SCORE	T-SCORE
AGE_MATCHED	Percentile in age-matched adults
Z_SCORE	Z-SCORE
