# Supplementary material for: AI-enhanced integration of genetic and medical imaging data for risk assessment of Type 2 diabetes
Source: Nat Commun. 2024 May 18;15:4230. doi: 10.1038/s41467-024-48618-1 (PMC11102564; doi:10.1038/s41467-024-48618-1)
Supplement: Supplementary file 5 — Reporting Summary [file 41467_2024_48618_MOESM5_ESM.pdf]

nature portfolio

Corresponding author(s): Hsin-Chou Yang

Last updated by author(s): April 19, 2024

Reporting Summary

Nature Portfolio wishes to improve the reproducibility of the work that we publish. This form provides structure for consistency and transparency in reporting. For further information on Nature Portfolio policies, see our Editorial Policies and the Editorial Policy Checklist.

Please do not complete any field with "not applicable" or n/a. Refer to the help text for what text to use if an item is not relevant to your study.
For final submission: please carefully check your responses for accuracy; you will not be able to make changes later.

Statistics

For all statistical analyses, confirm that the following items are present in the figure legend, table legend, main text, or Methods section.

n/a	Confirmed	
☐	☑	The exact sample size (*n*) for each experimental group/condition, given as a discrete number and unit of measurement
☐	☑	A statement on whether measurements were taken from distinct samples or whether the same sample was measured repeatedly
☐	☑	The statistical test(s) used AND whether they are one- or two-sided<br>*Only common tests should be described solely by name; describe more complex techniques in the Methods section.*
☐	☑	A description of all covariates tested
☐	☑	A description of any assumptions or corrections, such as tests of normality and adjustment for multiple comparisons
☐	☑	A full description of the statistical parameters including central tendency (e.g. means) or other basic estimates (e.g. regression coefficient) AND variation (e.g. standard deviation) or associated estimates of uncertainty (e.g. confidence intervals)
☐	☑	For null hypothesis testing, the test statistic (e.g. *F*, *t*, *r*) with confidence intervals, effect sizes, degrees of freedom and *P* value noted<br>*Give P values as exact values whenever suitable.*
☑	☐	For Bayesian analysis, information on the choice of priors and Markov chain Monte Carlo settings
☑	☐	For hierarchical and complex designs, identification of the appropriate level for tests and full reporting of outcomes
☑	☐	Estimates of effect sizes (e.g. Cohen's *d*, Pearson's *r*), indicating how they were calculated

Our web collection on statistics for biologists contains articles on many of the points above.

Software and code

Policy information about availability of computer code

Data collection	We did not collect and only analyzed the data collected by the Taiwan Biobank.
Data analysis	The code central to this study has been deposited to https://github.com/yjhuang1119/Risk-assessment-model.

For manuscripts utilizing custom algorithms or software that are central to the research but not yet described in published literature, software must be made available to editors and reviewers. We strongly encourage code deposition in a community repository (e.g. GitHub). See the Nature Portfolio guidelines for submitting code & software for further information.

Data

Policy information about availability of data

All manuscripts must include a data availability statement. This statement should provide the following information, where applicable:

- Accession codes, unique identifiers, or web links for publicly available datasets
- A description of any restrictions on data availability
- For clinical datasets or third party data, please ensure that the statement adheres to our policy

The data analyzed in this study were obtained from the Taiwan Biobank with proper approval. As the data are subject to ownership rights held by the Taiwan Biobank, they have not been deposited in a public repository. Researchers interested in accessing the data must do so through a formal application process, subject to approval by the Taiwan Biobank. Detailed instructions on requesting data access can be found on the Taiwan Biobank's official website (https://www.twbiobank.org.tw/index.php). Source data are provided in the Supplementary Information and Source Data files with this paper. In addition to the TWB data, a set of 137 highly significant T2D-associated SNPs from the AGEN can be downloaded from https://blog.nus.edu.sg/agen/summary-statistics/t2d-2020/. Meta-GWAS summary statistics of T2D in multiple populations from the DIAGRAM Consortium can be obtained from https://diagram-consortium.org/downloads.html. The linkage disequilibrium reference from multiple populations of the 1000 Genomes Project is available for download at https://github.com/getian107/PRScsx.

Research involving human participants, their data, or biological material

Policy information about studies with human participants or human data. See also policy information about sex, gender (identity/presentation), and sexual orientation and race, ethnicity and racism.

Reporting on sex and gender	Analyses and reports for males and females are included.
Reporting on race, ethnicity, or other socially relevant groupings	The study participants are the Han Chinese population in Taiwan.
Population characteristics	The study participants comprise the Han Chinese population in Taiwan, with a mean age of 50.57 (SD = 10.57) and 31.72% being male.
Recruitment	This study did not recruit participants and only analyzed data from the Taiwan Biobank and publicly available data.
Ethics oversight	The TWB collected written informed consent from all participants. The TWB (TWBR10911-01 and TWBR11005-04) and the Institute Review Board at Academia Sinica approved our data application and use (AS-IRB01-17049 and AS-IRB01-21009).

Note that full information on the approval of the study protocol must also be provided in the manuscript.

Field-specific reporting

Please select the one below that is the best fit for your research. If you are not sure, read the appropriate sections before making your selection.

☑ Life sciences ☐ Behavioural & social sciences ☐ Ecological, evolutionary & environmental sciences

For a reference copy of the document with all sections, see nature.com/documents/nr-reporting-summary-flat.pdf

Life sciences study design

All studies must disclose on these points even when the disclosure is negative.

Sample size	A total of 68,911 participants in the TWB were analyzed.
Data exclusions	Our analysis excluded participants who lacked TWB2.0 SNP array data.
Replication	A dataset consisting of N = 936 individuals, with both baseline and follow-up data, was later independently provided by the Taiwan Biobank. This analysis, involving N = 936, was exclusively conducted as a replication study within our research.
Randomization	Not available
Blinding	Not available

Behavioural & social sciences study design

All studies must disclose on these points even when the disclosure is negative.

Study description	Not available
Research sample	Not available
Sampling strategy	Not available
Data collection	Not available
Timing	Not available
Data exclusions	Not available
Non-participation	Not available
Randomization	Not available

Ecological, evolutionary & environmental sciences study design

All studies must disclose on these points even when the disclosure is negative.

Study description	Not available
Research sample	Not available
Sampling strategy	Not available
Data collection	Not available
Timing and spatial scale	Not available
Data exclusions	Not available
Reproducibility	Not available
Randomization	Not available
Blinding	Not available

Did the study involve field work? ☐ Yes ☑ No

Field work, collection and transport

Field conditions	Not available
Location	Not available
Access & import/export	Not available
Disturbance	Not available

Reporting for specific materials, systems and methods

We require information from authors about some types of materials, experimental systems and methods used in many studies. Here, indicate whether each material, system or method listed is relevant to your study. If you are not sure if a list item applies to your research, read the appropriate section before selecting a response.

Materials & experimental systems

n/a	Involved in the study	
☑	☐	Antibodies
☑	☐	Eukaryotic cell lines
☑	☐	Palaeontology and archaeology
☑	☐	Animals and other organisms
☑	☐	Clinical data
☑	☐	Dual use research of concern
☑	☐	Plants

Methods

n/a	Involved in the study	
☑	☐	ChIP-seq
☑	☐	Flow cytometry
☑	☐	MRI-based neuroimaging

Antibodies

Antibodies used	Not available
Validation	Not available

Eukaryotic cell lines

Policy information about cell lines and Sex and Gender in Research

Cell line source(s)	Not available
Authentication	Not available
Mycoplasma contamination	Not available
Commonly misidentified lines (See ICLAC register)	Not available

Palaeontology and Archaeology

Specimen provenance	Not available
Specimen deposition	Not available
Dating methods	Not available

☐ Tick this box to confirm that the raw and calibrated dates are available in the paper or in Supplementary Information.

Ethics oversight	Not available

Note that full information on the approval of the study protocol must also be provided in the manuscript.

Animals and other research organisms

Policy information about studies involving animals; ARRIVE guidelines recommended for reporting animal research, and Sex and Gender in Research

Laboratory animals	Not available
Wild animals	Not available
Reporting on sex	Not available
Field-collected samples	Not available
Ethics oversight	Not available

Note that full information on the approval of the study protocol must also be provided in the manuscript.

Clinical data

Policy information about clinical studies

All manuscripts should comply with the ICMJE guidelines for publication of clinical research and a completed CONSORT checklist must be included with all submissions.

Clinical trial registration	Not available
Study protocol	Not available
Data collection	Not available
Outcomes	Not available

Dual use research of concern

Policy information about dual use research of concern

Hazards

Could the accidental, deliberate or reckless misuse of agents or technologies generated in the work, or the application of information presented in the manuscript, pose a threat to:

No	Yes	
☑	☐	Public health
☑	☐	National security
☑	☐	Crops and/or livestock
☑	☐	Ecosystems
☑	☐	Any other significant area

Experiments of concern

Does the work involve any of these experiments of concern:

No	Yes	
☑	☐	Demonstrate how to render a vaccine ineffective
☑	☐	Confer resistance to therapeutically useful antibiotics or antiviral agents
☑	☐	Enhance the virulence of a pathogen or render a nonpathogen virulent
☑	☐	Increase transmissibility of a pathogen
☑	☐	Alter the host range of a pathogen
☑	☐	Enable evasion of diagnostic/detection modalities
☑	☐	Enable the weaponization of a biological agent or toxin
☑	☐	Any other potentially harmful combination of experiments and agents

Plants

Seed stocks	Not available
Novel plant genotypes	Not available
Authentication	Not available

ChIP-seq

Data deposition

☐ Confirm that both raw and final processed data have been deposited in a public database such as GEO.

☐ Confirm that you have deposited or provided access to graph files (e.g. BED files) for the called peaks.

Data access links<br>*May remain private before publication.*	Not available
Files in database submission	Not available
Genome browser session<br>(e.g. UCSC)	Not available

Methodology

Replicates	Not available
Sequencing depth	Not available
Antibodies	Not available
Peak calling parameters	Not available
Data quality	Not available
Software	Not available

Flow Cytometry

Plots

Confirm that:

☐ The axis labels state the marker and fluorochrome used (e.g. CD4-FITC).

☐ The axis scales are clearly visible. Include numbers along axes only for bottom left plot of group (a 'group' is an analysis of identical markers).

☐ All plots are contour plots with outliers or pseudocolor plots.

☐ A numerical value for number of cells or percentage (with statistics) is provided.

Methodology

Sample preparation	Not available
Instrument	Not available
Software	Not available
Cell population abundance	Not available
Gating strategy	Not available

☐ Tick this box to confirm that a figure exemplifying the gating strategy is provided in the Supplementary Information.

Magnetic resonance imaging

Experimental design

Design type	Not available
Design specifications	Not available
Behavioral performance measures	Not available

Imaging type(s)	Not available
Field strength	Not available
Sequence & imaging parameters	Not available
Area of acquisition	Not available

Diffusion MRI ☐ Used ☐ Not used

Preprocessing

Preprocessing software	Not available
Normalization	Not available
Normalization template	Not available
Noise and artifact removal	Not available
Volume censoring	Not available

Statistical modeling & inference

Model type and settings	Not available
Effect(s) tested	Not available

Specify type of analysis: ☐ Whole brain ☐ ROI-based ☐ Both

Statistic type for inference

(See Eklund et al. 2016)

Not available

Correction

Not available

Models & analysis

n/a	Involved in the study
✓	☐ Functional and/or effective connectivity
✓	☐ Graph analysis
✓	☐ Multivariate modeling or predictive analysis

Functional and/or effective connectivity

Graph analysis

Multivariate modeling and predictive analysis

This checklist template is licensed under a Creative Commons Attribution 4.0 International License, which permits use, sharing, adaptation, distribution and reproduction in any medium or format, as long as you give appropriate credit to the original author(s) and the source, provide a link to the Creative Commons license, and indicate if changes were made. The images or other third party material in this article are included in the article's Creative Commons license, unless indicated otherwise in a credit line to the material. If material is not included in the article's Creative Commons license and your intended use is not permitted by statutory regulation or exceeds the permitted use, you will need to obtain permission directly from the copyright holder. To view a copy of this license, visit http://creativecommons.org/licenses/by/4.0/
